# Usefulness of peripheral venous blood gas analyses in cats with arterial thromboembolism

**DOI:** 10.1080/23144599.2021.1982335

**Published:** 2021-10-26

**Authors:** Jidapa Tosuwan, Vachira Hunprasit, Sirilak Disatian Surachetpong

**Affiliations:** Department of Veterinary Medicine, Faculty of Veterinary Science, Chulalongkorn University, Bangkok Thailand

**Keywords:** Cat, arterial thromboembolism, venous blood gas analysis

## Abstract

Feline arterial thromboembolism (ATE) is a condition with a high mortality rate. Acid-base abnormalities may be beneficial to the prognosis of cats with ATE. Venous blood gas and electrolytes data on the first day of ATE presentation of 47 cats with ATE were retrospectively reviewed and analysed. The Cox and logistic regression were analysed to evaluate the relationship between acid-base parameters and death. The most common venous acid-base disorder was simple metabolic acidosis. Age, body weight, and partial venous pressure of carbon dioxide (PvCO2) differed between the dead and alive groups within 7 days of the onset of ATE presentation (*p* < 0.05). Cox-regression showed that increasing age (HR=1.175 [95% CI: 1.027-1.343], *p* = 0.019), increasing PvCO2 (HR=1.066 [95% CI: 1.010-1.125], *p* = 0.021) and PvCO2 more than 34 mmHg (HR=7.878 [95% CI: 1.036- 59.915], *p* = 0.046) were associated with increased hazard of death. Multivariable logistic regression showed that age > 5 years (OR=9.030, 95% CI: 1.258- 64.823; *p*=0.029), and PvCO2 > 34 mmHg (OR=21.764, 95% CI: 1.747-271.141; *p*=0.017) were associated with an increased risk of death, while concomitant administration of enoxaparin with clopidogrel (OR=0.111, 95% CI: 0.015-0.795; *p*=0.029) were associated with a decreased risk of death within 7 days of the onset of ATE presentation. This study demonstrated the power of venous blood gas analysis which may be used as prognostic indicators for cats with ATE.

## 1. Introduction

Feline arterial thromboembolism (ATE) is an acute and life-threatening condition when cats present to the emergency unit. Cats with ATE have a high mortality rate and poor prognosis. The survival rate to discharge in cats with ATE has been reported as 33–39% of cases [1–5]. The most common clinical signs include acute onset hindlimb paralysis, pulselessness, pain, poikilothermy and pallor of the extremities, typically affecting one or both hindlimbs. Some cats with ATE have additional clinical signs related to concurrent problems such as congestive heart failure or reperfusion injury. The majority of ATE are secondary to heart diseases and only a limited proportion to hyperthyroidism, neoplasm or coagulopathies diseases [6]. Rush and colleagues showed that the most common cause of death in cats with hypertrophic cardiomyopathy (HCM) was ATE, followed by congestive heart failure (CHF) and sudden cardiac arrest [7]. ATE cats may have biochemical changes such as elevated alanine aminotransferase, glucose and creatinine, and these laboratory changes may also provide information related to survival [8].

Acid-base disorders are frequently found in critically ill feline patients with diagnostic and prognostic relevance [8–10]. The association between acid-based disorders and mortality has been reported in various critical conditions in cats such as advanced chronic kidney disease and gastrointestinal obstruction [11–13]. Venous blood gas analyses are valuable tests for assessing acid-base status and measuring blood pH, bicarbonate (HCO_3_^−^) level, venous carbon dioxide level and anion gap (AG). Venous blood gases can typically be used to evaluate metabolic acidosis, a common acid-base disorder that has been reported in 49% of dogs and cats [14].

The identification of acid-base disorders is likely to be a valuable tool for the effective detection and treatment of metabolic disorders in critically ill animals. Considering that information on the usefulness of venous blood gas analysis in cats with ATE is currently sparse, whether acid-base balance assessment can help clinicians in the clinical management of affected cats and whether it can provide prognostic information remains to be conclusively determined. Therefore, the present study aimed to investigate venous acid-base alterations associated with feline ATE and their association with sudden death in a retrospective study of 47 cats with ATE.

## 2. Materials and methods

### 2.1. Ethical statement

The study was conducted at Faculty of Veterinary Science, Chulalongkorn University, Thailand. Ethical approval was not required for this study because of its retrospective design. The database assessment was approved by the Small Animal Teaching Hospital Board committees.

### 2.2. Sample collection

Blood gas analysis of cats diagnosed with ATE during 2017–2020 at the Small Animal Teaching Hospital, Faculty of Veterinary Science, Chulalongkorn University, Thailand was retrospectively retrieved from medical records. Venous blood gas analysis was measured on the first arrival of cats presenting with clinical signs of ATE without having received prior treatment. Included cats were required to arrive at the hospital within 24 hours of first presentation of ATE signs. Heparinized blood samples for acid-base parameters and electrolyte concentrations were measured immediately after sample collection using a point-of-care device (RAPIDLab® 348, SIEMENS, Erlangen, Germany).

The diagnosis of ATE was based on history and clinical signs of limb paralysis with pulselessness, pain, poikilothermy and pallor of the extremities at presentation [3,15]. A Doppler device was used to find the pulse on the affected limbs. If deemed clinically necessary, ultrasonography was performed to confirm the presence of arterial occlusion at the aortic trifurcation. Both cardiogenic and non-cardiogenic ATE were included. Cardiogenic causes were congenital and acquired cardiac diseases [2,16]. Cats with and without concurrent congestive heart failure (CHF) were all included.

### 2.3. Variables

Blood gas and electrolyte variables included pH, partial venous pressure of carbondioxide (PvCO_2_), bicarbonate (HCO_3_^−^), sodium (Na), potassium (K), chloride (Cl) concentrations, haematocrit, and anion gap.

Acid-base disorders were approached using traditional acid-base analysis and classified using the criteria described by Hopper et al. [17].

### 2.4. Statistical analysis

Initial descriptive statistics included mean ± standard deviation for normally distributed data and median and interquartile range (IQR) for non-normally distributed data. The Kolmogorov–Smirnov test was used to test the normality of the data. Venous blood gas measurements were compared with the reference range in normal cats [17]. Alive or dead status of cats on day 7 after ATE presentation was recorded and used for blood gas analysis, Cox-regression and logistic regression. The timing for the day 7 analysis was chosen to follow a previous study [15]. The venous blood gas was analysed using the traditional acid-base diagnostic method. Student’s t-test or Mann-Whitney U test was used to compare the numerical data between the alive and dead groups. A *p-value* of < 0.05 was considered significant.

Cox-regression was applied to identify the hazard risk factors associated with survival time in cats with ATE. Survival time was recorded as the period from the first day of the initial presentation of ATE signs to the day of death. Cats with ATE, which died within the first 7 days of ATE presentation, were used as status. A *p*-value of <0.05 was considered significant. Kaplan-Meier method was used to estimate the probability of survival within 7 days of the onset of ATE presentation based on the survival probability of the hazard risk factor of interest.

Logistic regression was performed to develop the prediction model of death within 7 days after the onset of ATE presentation. For clinical relevance, we used the cut-off value for PvCO_2_ of 34 mmHg, which was the median PvCO_2_ of cats presenting to the emergency unit [17]; an age of 5 years, which was the age that influenced the survival of cats with ATE [18]; and a body weight of 3.5 kg, which represented the median body weight of cats typically referred in our country according to previous publications [19,20]. All categorical factors that had a *p-*value of > 0.2 assessed by the univariate logistic regression were subjected to multiple logistic regression. Interaction and multicollinearity between parameters were assessed. The backward Wald elimination method was used to create the final model. The model that met the majority of the statistical model consumptions with the largest Nagelkerke r^2^ was selected. The goodness of the statistical model assumption included: the overall percentage from the classification table was >70%, the Hosmer-Lemeshow test was not significant (*p > *0.05) and the area under the receiver operating characteristic curve was > 0.80 [21]. A *p*-value of <0.05 was considered significant. The commercial software SPSS version 22 (Inc, Chicago, IL, USA.) was used for statistical analysis.

## 3. Results

This retrospective study included 47 cats with ATE presented to the Small Animal Teaching Hospital, Faculty of Veterinary Science, Chulalongkorn University, Thailand during 2016–2020. The study included 25 males (53.2%) and 22 (46.8%) females. Cat breeds included 37 domestic shorthairs (78.7%), 7 Persians (14.9%), 2 Maine Coons (4.3%) and 1 British Shorthair (2.1%). In twelve cats, the presence of the arterial occlusion was confirmed at aortic trifurcation by ultrasonography. Thirty-six cats (76.6%) were diagnosed with cardiomyopathy assessed by echocardiography, including hypertrophic cardiomyopathy (31/36, 86.11%) and restrictive cardiomyopathy (RCM) (5/36, 13.89%). On electrocardiography, 6/27 cats had arrhythmias including atrial fibrillation (3/6, 50%), ventricular premature complex (2/6, 33.3%) and mixed atrial fibrillation with ventricular premature complex (1/6; 16.7%). All 6 cats with arrhythmias had underlying heart diseases including hypertrophic cardiomyopathy (3/6) and restrictive cardiomyopathy (3/6). In addition, 23/36 (63.8%) cats had CHF (20 cats had pulmonary oedema and 3 cats had both pulmonary oedema and pleural effusion).

Ten cats (21.3%) had normal cardiac structure on echocardiography. One cat did not have echocardiography. Three out of 10 cats with non-cardiogenic ATE, showed ATE signs after ovariohysterectomy. Three out of 10 cats had concurrent illnesses including sepsis, *Mycoplasma haemofelis* infection and feline leukaemia virus infection. No obvious diseases were noted in the other 4 cats with non-cardiogenic ATE.

Twenty-six cats (55.3%) were hospitalized for an average of 6.3 days. Forty-one cats (87.2%) were anorectic and received fluid supplementation with Acetate’s Ringer solution, 32 cats (78%) received fluid intravenously, and 9 cats (22%) received fluid subcutaneously. The rate and volume of fluid therapy depended on hydration status and individual requirement. Eight cats received oxygen supplementation via oxygen cage during hospitalization. Twenty-one cats with CHF were supplemented with fluid after CHF was controlled with cardiovascular medications because the cats still could not drink well. The total volume of fluid therapy was given within one-third of the daily fluid requirement in cats with CHF. The fluid supplementation was stopped as soon as the cats were able to drink properly. Twenty-nine cats (61.7%) received analgesics including tramadol (21/29, 72.4%) at dose of 4 mg/kg subcutaneously q8-12 h, morphine (6/29, 20.8%) at a dose of 0.2 mg/kg subcutaneously q12h, fentanyl 25 μg/h transdermal patch (1/29, 3.4%) and gabapentin (1/29, 3.4%) at a dose of 5 mg/kg PO q12h.

Forty-six cats (97.8%) received anticoagulants and antiplatelet agents including enoxaparin 1 mg/kg subcutaneously q6-12 h and clopidogrel 18.75 mg per cat PO q24h. Twenty-four cats (52.2%) received enoxaparin with clopidogrel, 13 cats (28.2%) received enoxaparin only and 9 cats (19.6%) received clopidogrel only. Twenty-two cats (46.8%) received furosemide (2 mg/kg intravenously or orally q8-12 h) for the treatment of CHF. One cat received no medication due to the owner decision. None of the cats in this study received oxygen supplementation, fluid therapy, or medication prior to venous blood gas sampling for blood gas analysis.

On day 7, 22/39 (56.4%) cats were alive and 17/39 (43.6%) cats died. Only 13 cats that died had specified causes of death including CHF (4/13; 30.8%), euthanasia (1/13; 7/7%), and sudden cardiac death presumably caused by reperfusion injury (8/13; 61.5%).

The median age, body weight, day of survival, acid-base and electrolyte levels on the first day of ATE presentation of 47 cats are shown in Table 1. In this study, cats with ATE had median values of pH, Na, K, Cl, and HCO_3_^−^ that were below than the normal reference range.

**Table 1.** Venous blood gas analysis of 47 cats with arterial thromboembolism (ATE)

**Parameters**	**Median (Q1 – Q3)**	**Reference [**14**]**
Age (years)	4 (2–7)	
Bodyweight (kg)	4.35 (3.4–4.9)	
Survival day (days)	7 (3–202)	
pH	7.33 (7.276–7.374)	7.34–7.43
PvCO_2_ (mmHg)	35.6 (31.7–39.8)	34–39
Sodium (mmol/L)	139 (134–145)	148–156
Potassium (mmol/L)	3.31 (2.79–4.26)	3.4–4.7
Chloride (mmol/L)	106 (100–114)	115–126
HCT	40 (36–44)	30–44
HCO_3_^−^ (mmol/L)	18.8 (15.7–20.6)	20–23
AG	17.2 (8.9–27.1)	16–20

AG: anion gap; HCO_3_^−^: bicarbonate; HCT: haematocrit; PvCO_2_: venous partial pressure of CO

Traditional venous acid-base analysis showed an abnormality in 30 cats with ATE. Seventeen cats had normal acid-base results, 2 cats had simple respiratory acid-base abnormalities, 12 cats had metabolic acid-base abnormalities, and 7 cats had mixed abnormalities (Table 2). The most common abnormality was simple metabolic acidosis followed by respiratory acidosis.

**Table 2.** Traditional acid–base diagnosis of 47 cats with arterial thromboembolism (ATE)

Acid–base disorder	Number of cats (n = 47)
Normal acid–base	17 (36.2%)
**Simple disorder**	
Respiratory acidosis	7 (14.9%)
Respiratory alkalosis	4 (8.5%)
Metabolic acidosis	12 (25.5%)
with normal AG	5 (10.6%)
with increased AG	7 (14.9%)
**Mixed disorder**	
Metabolic acidosis with respiratory acidosis	1 (2.1%)
Metabolic acidosis with respiratory alkalosis	6 (12.8%)
Total	47 (100%)

AG: anion gap

The medians of age, body weight, acid-base and electrolyte levels on the first day of ATE presentation of 39 cats divided into 2 groups: the dead and alive groups within seven days of ATE presentation are shown in Table 3.

**Table 3.** Comparisons of median (IQR) between the dead and alive groups at day 7 after the arterial thromboembolism (ATE) presentation of 39 cats

**Parameter**	**Dead (n = 17/39)**	**Alive (n = 22/39)**	**Reference [**14**]**	*p-*value*
Age (years)	5 (3–9)	2.50 (1.75–5.00)		0.006
Body weight (kg)	4.80 (4.10–5.12)	4.125 (3.08–4.73)		0.039
pH	7.33 (7.27–7.37)	7.35 (7.30–7.40)	7.34–7.43	0.221
PvCO_2_ (mmHg)	36.70 (35.05–41.55)	33 (31.13–38.70)	34–39	0.034
PvO_2_ (mmHg)	38.50 (29.80–43.45)	37.15 (28.23–48.43)	-	0.900
Sodium (mmol/L)	141 (135.5–144.5)	138.5 (133–142)	148–156	0.377
Potassium (mmol/L)	3.59 (2.96–4.31)	3.015 (2.69–3.69)	3.4–4.7	0.081
Chloride (mmol/L)	107 (100–115.5)	102.5 (97.75–109)	115–126	0.104
HCT	40 (36.5–41)	43 (33–46.25)	30–44	0.812
HCO_3_^−^ (mmol/L)	18.9 (17.65–20.6)	18.3 (15.38–20.35)	20–23	0.232
Anion gap	12.3 (7.65–26.95)	20.35 (14.8–27.38)	16–20	0.243

*The *p*-value <0.05 represent the significant difference between the dead and alive groups by

the Mann-Whitney U test

AnGap: anion gap; HCO_3_^−^: bicarbonate; HCT: haematocrit; PvCO_2_: venous partial pressure of CO; PvO_2_: venous partial pressure of oxygen

**Figure1.**
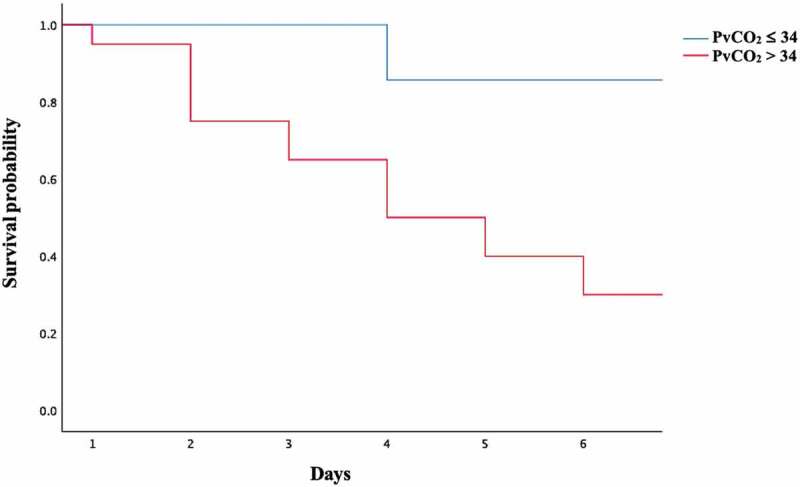
Kaplan Meier graphs compare survival time at day 7 after arterial thromboembolism (ATE) presentation between PvCO_2_ < 34 (blue line) and PvCO_2_ > 34 (red line)

Univariate Cox-regression was used to identify the hazard risk factor associated with death of 27 cats that died within 7 days of ATE presentation. Significant factors included increased age (HR = 1.175 [95% CI: 1.027–1.343], *p* = 0.019), increased PvCO_2_ (HR = 1.066 [95% CI: 1.010–1.125], *p* = 0.021) and PvCO_2_ > 34 mmHg (HR = 7.878 [95% CI: 1.036–59.915], *p* = 0.046). Kaplan-Meier Survival plots of cats with PvCO_2_ < 34 mmHg and PvCO_2_ > 34 mmHg at day 7 after the onset of ATE presentation are shown in Figure 1.

Univariate logistic regression showed that the significant factors for death of cats within 7 days after ATE presentation were age, specially > 5 years old; increased PvCO_2_, specially > 34 mmHg; increased body weight, specially > 3.5 kilograms; and combination treatment of enoxaparin and clopidogrel. Age of > 5 years old, PvCO_2_ > 34 mmHg and combination treatment of enoxaparin and clopidogrel were able to predict the risk of death within 7 days after the onset of ATE presentation from the multivariable logistic regression (Table 4).

**Table 4.** Risk factors related to death within 7 days after the onset of arterial thromboembolism presentation

Variables	Univariate Logistic Regression		Multiple Logistic Regression ^a^	
	Crude OR (95% CI)	*p-*value	Adjusted OR (95% CI)	*p-*value
Age	1.243 (1.008–1.532)	0.042		
Age>5	4.889 (1.246–19.190)	0.023	9.030 (1.258–64.823)	0.029
PvCO_2_	1.174 (1.005–1.371)	0.043		
PvCO_2_ > 34	19.20 (2.154–171.154)	0.008	21.764 (1.747–271.141)	0.017
Bodyweight	2.360 (1.043–5.338)	0.039		
Bodyweight>3.5	9.143 (1.014–82.442)	0.049		
Coadministration of enoxaparin and clopidogrel	0.205 (0.052–0.803)	0.023	0.111 (0.015–0.795)	0.029

CI: confident interval; OR: odd ratio

^a^Hosmer-Lemeshow test (*p >* 0.05), overall percentage from classification table = 82.1%, area under the ROC curve = 0.876 and Nagelkerke r^2^ = 0.576.

## 4. Discussion

The present study documented venous acid-base levels associated with ATE and their association with death in a retrospective study of 47 cats with ATE. Cats with other critical illnesses may have abnormalities in oxygenation, ventilation, electrolytes and acid-base balance [11–13,22]. Evaluation of these parameters should be recognized to detect the life-threatening abnormalities in cats with ATE. To our knowledge, this is the first study reporting the venous blood gas in cats with ATE.

The majority of cats in this study were domestic shorthair, similar to previous studies [1,2,18]. This finding may reflect the high proportion of this breed in Bangkok [23], not a true intrinsic predisposition of this breed to ATE. There was no difference in the incidence of ATE in males and females in this study. The majority of cats (76%) had cardiogenic ATE. The main cause of cardiogenic ATE was hypertrophic cardiomyopathy (86%), similar to a previous study [18]. This result is probably due to the fact that HCM is the leading cause of heart disease in cats in Thailand [18] and other countries around the world [24]. Other causes of ATE in this study could be related to postoperative thrombosis, immune-mediated haemolytic anaemia due to *Mycoplasma haemofelis* [25] and severe inflammation secondary to septic state [26]. The underlying cause was not identified in 4 cats with non-cardiogenic ATE in the present study. The predisposing cause of ATE may be missed in some cats subjected to antemortem diagnostic testing [2]. Therefore, a post-mortem diagnosis should be performed to identify the underlying diseases causing ATE. All cardiogenic ATE cats with cardiac arrhythmias died within 7 days. This finding was consistent with previous studies that HCM and RCM cats with arrhythmia have a worse prognosis [27–29]. Approximately half of the cats affected with ATE in this study had concurrent CHF similar to other studies that reported CHF in 44–66% of cases with ATE [1–3].

The median pH, Na, Cl, K, and HCO_3_^−^ concentrations of the cats in this study were below normal limits. The pH indicates the acid-base status of a patient. Acidosis occurs when the pH is below the normal limit. This condition involves pathophysiological processes that cause net accumulation of acid in the body due to decreased plasma HCO_3_^−^, increased plasma CO_2_ or both. In our study, the median HCO_3_^−^ concentration was lower than the normal limit, indicating the tendency towards metabolic acidosis, which may be caused by lactic acid accumulation secondary to tissue ischaemia and hypoxia in the ATE affected limbs [30,35].

Sodium is present as charged particles in the aqueous phase of body fluids. A probable likely cause of hyponatraemia in the present study is concurrent CHF. The dilution effect may result from markedly increased fluid accumulation in the circulation, probably mediated by nonosmotic release of arginine vasopressin (ADH) in response to inadequate cardiac output [30]. Serum Cl concentration can be altered by water balance (i.e. an increase or decrease in free water) or by a gain or loss of Cl. Changes in plasma Cl and Na concentrations are proportional. To account for changes in water balance, Cl concentration must be evaluated in conjunction with changes in Na concentration; this approach permits the division of Cl disorders into artifactual and corrected changes. The corrected Cl concentration is calculated as Cl x 156/Na. The normal corrected Cl concentration in cats is approximately 117–123 mEq/L [31]. From our results, the median corrected Cl concentration was 119 mEq/L, which is within the normal range. While Na is the major extracellular cation, K is the major intracellular cation in mammalian cells. In cats, the total body K concentration is about 55 mEq/kg body weight. Approximately 95% or more of the total body K is in the intracellular fluid (ICF) while the remaining 5% is in the extracellular fluid (ECF). During translocation of K between the ICF and the ECF, serum K concentration may change without alterations in total body K content. Hypokalemia is associated with decreased K intake, translocation of K between the ICF and the ECF, and increased K loss. Anorexia is common in patients with thromboembolism and is associated with hyperaesthesia and/or CHF [31]. Hypokalemia in cats with ATE may occur secondary to decreased K intake from anorexia. Hyperkalaemia may occur in cats with ATE secondary to an exchange of K+ and H+ in a state of acidosis or reperfusion injury [30]. None of the cats in this study had hyperkalaemia (K+>5.5 mEq/L) because hyperkalaemia is not common in the acute phase of ATE but may be developed during treatment from reperfusion of ischaemic hindlimb muscles. Reperfusion syndrome is found in 40–70% of cats after thrombolytic therapy and is the most common cause of death [14].

The alive or dead status of cats on day 7 after ATE presentation was reported and used for blood gas analysis, Cox-regression and logistic regression. This time point was chosen for two reasons: (1) the median survival time of cats with ATE in this study was approximately 7 days and (2) in a previous study, the authors found numerous predictors of mortality within 7 days of ATE presentation but no significant predictors after 7 days [15].

Approximately one-third of the cats with ATE in this study had normal acid-base balance on the first day of ATE presentation, but nearly half of them died within 7 days. The most common acid-base disorder in cats in this study was simple metabolic acidosis, which is the most common acid-base disorder in critically ill cats and dogs [14]. Metabolic acidosis is associated with several diseases and conditions. Metabolic acidosis with an increased anion gap can be caused by tissue ischaemia of ATE or stagnant circulation with lactic acidosis and hypoxia, which can occur in cats with CHF [30,32]. In contrast, metabolic acidosis with a normal anion gap can be caused by post-hypocapnia due to hyperventilation from acute and severe pain during the onset of ATE [32]. Respiratory acidosis was the second most common acid-base disorder in this study. Respiratory acidosis is a more serious complication and indicates the presence of respiratory insufficiency which may occur secondary to pulmonary oedema. Respiratory acidosis may also occur due to decreased alveolar ventilation caused by neuromuscular abnormalities associated with hypokalemia (K+ <3 mEq/l) and depression of the respiratory centre induced by narcotics, namely morphine and tramadol, which were used as analgesics in this study [33,34].

Cats that died within 7 days of ATE presentation were older, heavier, and had higher PvCO_2_ compared with those that were alive, suggesting that cats with these characteristics have a poor prognosis. Similar findings have been reported in previous studies, which found that older cats with ATE had an increased risk of death [2,36]. Older cats may have concurrent or covert age related diseases that may affect mortality and prognosis. In human medicine, the association between obesity and thrombosis has been mentioned previously [37]. Chronic inflammation and impaired fibrinolysis have been proposed as major mechanisms of thrombosis in obese people [38]. However, the relationship between body weight and survival in cats with ATE has never been reported. Electrolytes between the dead and alive groups were not different. There was an increased hazard of death within 7 days of ATE presentation in cats with older age and elevated PvCO_2_, particularly in cats with PvCO_2_ > 34 mmHg, which had an 8-fold increased hazard of death. PvCO_2_ > 34 mmHg may indicate severe conditions such as respiratory muscle fatigue or respiratory failure [32], which may affect survival in cats with ATE. This finding suggests that PvCO_2_ from venous blood gas analysis may be useful in predicting the prognosis of cats with ATE.

Univariate logistic regression showed that older, heavier cats with higher PvCO_2_ had a higher risk of dying from ATE, whereas co-administration of enoxaparin and clopidogrel reduced the risk of death for cats with ATE. Cut-offs of > 5 years old of age, a PvCO_2_ of > 34 mmHg, and a body weight of > 3.5 kg were chosen for clinical use. Multivariable logistic regression showed that age > 5 years and PvCO_2_ > 34 mmHg were associated with an increased risk of death (5-and 19-fold, respectively) within 7 days of ATE, with cats aged > 5 years having shorter survival [18], indicating a better prognosis in younger cats developing ATE. Elevated PvCO_2_ from venous blood gas analysis indicated a worse prognosis. In addition, administration of enoxaparin and clopidogrel in combination may increase survival in cats with ATE by reducing thrombus formation through the dual action of the anticoagulants of enoxaparin and antiplatelet agents of clopidogrel [39]. Interestingly, the additional administration of either enoxaparin or clopidogrel alone was not associated with a reduced risk of death in cats with ATE in this study. This is the first time the concomitant administration of the two drugs has been shown to be statistically superior to clopidogrel or enoxaparin alone in terms of survival.

This study had some limitations. Venous samples for blood gas analysis were obtained from peripheral vessels of unaffected limbs in conscious, restrained cats. Therefore, possible stress induced fluctuations may occur. Hyperventilation may be related not only to acidosis resulting from ATE but also to stress and fear from restraint. Another limitation was the retrospective design of the study. Missing data may influence the statistical analysis.

## 5. Conclusion

This study demonstrated the association between venous blood gas analysis and prognosis in cats with ATE. The novel baseline blood gas analysis of cats with ATE was presented. One-third of cats with ATE had normal acid-base balance, whereas metabolic acidosis was the most common acid-base disorder. Cats with ATE with PvCO_2_ > 34 mmHg had an 8- fold increased hazard of death within 7 days of ATE presentation compared with cats with PvCO_2_ < 34 mmHg. Age > 5 years, and PvCO_2_ > 34 mmHg were associated with an increased risk of death, whereas concomitant administration of enoxaparin and clopidogrel was associated with a decreased risk of death within 7 days of ATE presentation. This study found that age and PvCO_2_ from venous blood gas analysis may be used as prognostic indicators for cats with ATE.
